# Editorial: Case reports in heart surgery: 2023

**DOI:** 10.3389/fcvm.2024.1527906

**Published:** 2024-12-09

**Authors:** Giuseppe Gatti

**Affiliations:** Cardiac Surgery Unit, Cardio-Thoracic and Vascular Department, Azienda Sanitaria Universitaria Giuliano-Isontina (ASUGI), Trieste, Italy

**Keywords:** myxoma, infective endocarditis, minimally invasive, multimodal imaging, transcatheter valve implantation, case report, heart surgery

**Editorial on the Research Topic**
Case reports in heart surgery: 2023

I would like to state from the beginning that I am sincerely grateful to the Editors-in-Chief of Frontiers in Cardiovascular Medicine and Frontiers in Surgery for having asked me to coordinate the 2023 collection of “*Cases Reports in Heart Surgery*”. I would also like to thank all the members of the Editorial Offices of the two valuable scientific Journals for having supported me at every moment of this experience. I hope I have been up to my task.

The objective of the present collection was to feature unique cases of patients that present with an unexpected diagnosis, treatment outcome, or clinical course. Only original Case Reports that significantly will advance the cardiac surgical field—at least in my opinion and that of the Reviewers who collaborated step by step with me in the manuscript review process—have been considered. Rare cases with typical features, frequent cases with atypical features, as well as cases with a convincing response to new treatments, were included in the present Research Topic.

The collection consists of 14 articles written by a total of 88 Authors from six countries (Canada, China, Germany, Iran, Spain and Switzerland) on three different continents. To date there have already been almost 13,000 total views.

Personally, I am particularly fond of the Case Reports sections of surgical Journals because they often include interesting and innovative contributions. The clinical presentation, diagnostic process and effective surgical treatment of rare conditions offer the reader stimulating food for thought. Sometimes there are reported cases of failure but of great educational value. However, Case Reports sections are increasingly rare nowadays in scientific Journals where more value is placed on large-scale studies such as multicenter studies, randomized controlled trials or meta-analyses. For all these reasons, and to give clear objectives and more relevance to Case Reports sections, Frontiers has introduced these regular collections of original surgical cases. I think this editorial initiative is worthy and, personally, I am flattered by the invitation to coordinate it. Both for Heart Surgery and Interventional Cardiology, the most advanced frontiers of the disciplines are often glimpsed by analyzing Case Reports!

Very current issues are addressed in the present collection. These issues can be summarized as follows:

- The growing importance of minimally invasive surgery and interventional techniques and technologies (1, 3, 5, 14), and of their complications [1, 3, 14];
- The essential need to carefully plan the surgical strategy before operation [1, 6, 8, 12];
- The essential need of a multimodal imaging for complex lesions [1, 3, 6, 7, 8, 10, 12, 13];
- The need to develop specific surgical techniques for the treatment of infective endocarditis [11];
- The unusual presentations of “usual” lesions or complications following traditional heart surgery [2, 4, 7, 9, 10, 12, 13].

I synthesized the main message of each contribution to the present collection in Table 1.

**Table 1:** Case reports in heart surgery 2023^a^.

	First author	Title	Publication date	DOI	Total views at 14 November 2024	Main message	Key words
1.	Caneiro-Queija et al.	Left ventricular outflow tract obstruction after transcatheter mitral valve replacement: a case report with a multifaceted approach	Aug 21, 2024	doi.org/10.3389/fcvm.2024.1431639	856	Successful management using medical treatment and alcohol septal ablation of LVOT obstruction following TMVR. Authors discuss about this event and the strategies to prevent and manage the condition.	Alcohol septal ablation; LVOT obstruction; TMVR
2.	Wang et al.	Case Report: Left atrial dissection after mitral valve replacement: intraoperative management under TEE guidance	Aug 5, 2024	doi.org/10.3389/fcvm.2024.1413713	812	A successful TEE-guided surgical treatment of LA dissection following MV surgery. Indications and timing of surgery are discussed by the Authors.	LA dissection; MV replacement; TEE
3.	Pommert et al.	TMVR after TA-TAVR: a re-redo surgery—case report	May 20, 2024	doi.org/10.3389/fcvm.2024.1373840	1,032	A successful TMVR for mitral regurgitation early after transcatheter mitral valve repair in a patient undergone previously to two cardiac operations, TA-TAVR and CABG. The Authors emphasize the role of CT imaging in predicting both interaction between the prosthetic valves, and anatomy of the neo-LVOT.	Cardiac reoperation; CT imaging; LVOT; TA-TAVR; TMVR
4.	Sharifkazemi et al.	Retrosternal hematoma causing torsade de pointes after coronary artery bypass graft surgery; a case report	May 20, 2024	doi.org/10.3389/fcvm.2024.1331873	816	The Authors analyze a case of malignant arrhythmia refractory to medical treatment and due to a post-CABG retrosternal hematoma.	Arrhythmia; CABG; Retrosternal hematoma
5.	Wang et al.	Case Report: Totally endoscopic minimally invasive mitral valve surgery during pregnancy: a case series	Feb 26, 2024	doi.org/10.3389/fcvm.2024.1300508	1,445	A nice series of pregnant women with MV diseases were successful treated using a totally endoscopic approach.	MV surgery; Minimally invasive surgery; Pregnancy;
6.	Khorgami et al.	Missile embolism from pulmonary vein to left ventricle: report of a case	Feb 23, 2024	doi.org/10.3389/fcvm.2024.1342146	969	Successful removal of a bullet, which was embolized from the pulmonary veins into the left ventricle of a child is reported.	Bullet; Embolism; Pediatric cardiac surgery
7.	Xiao et al.	Case Report: Giant left atrial cystic tumor: myxoma or intracardiac blood cyst?	Feb 14, 2024	doi.org/10.3389/fcvm.2024.1323890	1,816	Successful surgical removal of a rare case of giant LA cystic myxoma.	Cystic myxoma; Blood cyst; LA
8.	Buech et al.	Case Report: Incidental finding of an atresia of the inferior vena cava—a challenge for cardiac surgery	Feb 6, 2024	doi.org/10.3389/fcvm.2024.1321685	857	Perioperative management of IVC atresia during cardiopulmonary bypass. Preoperative diagnostics and intraoperative cannulation strategies to optimize venous drainage.	IVC atresia; Cannulation strategies; Cardiopulmonary bypass
9.	Tang et al.	Case Report: Acute cerebral infarction caused by left atrial and right ventricular myxoma received emergency operation	Jan 12, 2024	doi.org/10.3389/fcvm.2023.1316063	1,218	Insights from a rare case of ischemic stroke due to left atrial and right ventricular multiple cardiac myxoma.	Ischemic stroke; Multiple cardiac myxoma
10.	Wu et al.	Case Report: Surgical management of idiopathic pulmonary aneurysms and review surgical approaches	Dec 20, 2023	doi.org/10.3389/fcvm.2023.1331982	1,885	Following the treatment of a patient with idiopathic pulmonary aneurysm, and by reviewing most of recent surgical strategies, the Authors have developed an original treatment flow chart for this challenging disease. The flowchart, which serves as a guide for the management of idiopathic pulmonary aneurysm, offers valuable insights and evidence-based recommendations. A special issue is the optimal approach for addressing the main pulmonary artery, its branches, and the pulmonary valve.	Flow chart; Idiopathic pulmonary aneurysm; Surgical strategies
11.	Chu et al.	Case Report: Pericardial patch repair of mitral annulus and mitral valve for a left atrial dissection caused by parasitic infective endocarditis	Nov 28, 2023	doi.org/10.3389/fcvm.2023.1239019	913	A complex mitral valve repair using a patch of autologous pericardium was performed to treat a rare case of left atrial dissection due to parasitic infectious endocarditis.	Autologous pericardium; MV repair; Parasitic infective endocarditis
12.	Jolou et al.	Case Report: Right atrial mass arising from the Eustachian valve	Nov 9, 2023	doi.org/10.3389/fcvm.2023.1268918	1,773	An organized thrombus attached to the Eustachian valve and Chiari network was found 18 months after a cardiac operation. After performing an interesting discussion on the possible etiology of the mass, the Authors emphasize the need, in this case, of a surgical re-exploration to perform diagnosis.	Cardiac thrombosis; Chiari network; Eustachian valve; Surgical re-exploration
13.	Zhou et al.	Case Report: Myocardial dissection caused by ruptured sinus of Valsalva aneurysm in association with a bicuspid aortic valve	Nov 8, 2023	doi.org/10.3389/fcvm.2023.1289624	1,139	The Authors present an interesting case of myocardial dissection due to rupture of a Valsalva sinus aneurysm. The presence of a bicuspid aortic valve may be a predisposing factor, as well as favor suspicion and early detection using multimodal imaging.	Bicuspid aortic valve; Multimodal imaging; Myocardial dissection; Sinus of Valsalva rupture
14	Wang et al.	Case report: pulmonary artery perforation during transseptal puncture for left atrial appendage closure requires emergency cardiac operation	Oct 10, 2023	doi.org/10.3389/fcvm.2023.1218582	1,769	The Authors review a case of successful rescue surgery for pulmonary artery perforation during percutaneous LA appendage closure. The causal mechanisms, clinical presentation and all possible management strategies are carefully discussed.	Percutaneous LA appendage closure; Pulmonary artery perforation; Rescue cardiac surgery

CABG, coronary artery bypass grafting; CT, computed tomography; DOI, digital object identifier; IVC, inferior vena cava; TA-TAVR, transapical transcatheter aortic valve replacement; TEE, transesophageal echocardiography; TMVR, transcatheter mitral valve replacement; LA, left atrial/atrium; LVOT, left ventricular outflow tract.

^a^ Case Reports are reported according to decrescent publication date.

To conclude, I would like to sincerely thank all the valuable Reviewers and Co-editors who helped me in my task. I have certainly learned a lot from them throughout this experience.

